# Volatile Decay Products in Breath During Peritonitis Shock are Attenuated by Enteral Blockade of Pancreatic Digestive Proteases

**DOI:** 10.1097/SHK.0000000000000888

**Published:** 2017-10-16

**Authors:** Frank A. DeLano, Jason Chow, Geert W. Schmid-Schönbein

**Affiliations:** Microcirculation Laboratory, Department of Bioengineering, Institute for Engineering in Medicine, University of California San Diego, La Jolla, California

**Keywords:** Breath, pancreatic digestive enzymes, peritonitis shock, volatile organic compounds

## Abstract

There is a need to develop markers for early detection of organ failure in shock that can be noninvasively measured at point of care. We explore here the use of volatile organic compounds (VOCs) in expired air in a rat peritonitis shock model. Expired breath samples were collected into Tedlar gas bags and analyzed by standardized gas chromatography. The gas chromatograms were digitally analyzed for presence of peak amounts over a range of Kovach indices. Following the induction of peritonitis, selected volatile compounds were detected within about 1 h, which remained at elevated amounts over a 6 h observation period. These VOCs were not present in control animals without peritonitis. Comparisons with know VOCs indicate that they include 1,4-diaminobutane and trimethylamine *N*-oxide. When pancreatic digestive proteases were blocked with tranexamic acid in the intestine and peritoneum, a procedure that serves to reduce organ failure in shock, the amounts of VOCs in the breath decreased spontaneously to control values without peritonitis. These results indicate that peritonitis shock is accompanied by development of volatile organic compounds that may be generated by digestive enzymes in the small intestine. VOCs may serve as indicators for detection of early forms of autodigestion by digestive proteases.

## INTRODUCTION

One of the major requirements for effective intervention in shock is early detection of signs for progression toward organ failure. There are a large number of blood indicators for shock which range from monitoring of small molecules, like blood oxygen values, to detection of elevated blood plasma values of lactate or inflammatory markers such as complements and cytokines (1–3). Selected combinations of lower molecular weight (up to1.5 kD) metabolomic markers (e.g., organic acids, lipids, amino acids, carbohydrates, peptides, vitamins, steroids, xenobiotics) in exhaled breath condensates were proposed to characterize patients with shock and respiratory failure (4). But there is a need to explore noninvasive early indicators for end organ damage that serve to minimize delay in diagnosis and facilitate interventions to attenuate progression into multi-organ failure.

Volatile organic compounds (VOCs) have been identified as possible approaches for noninvasive detection of several diseases, e.g., cancers, acute lung injury, tuberculosis, liver disease, myocardial infarction (5–11). VOCs include acetone, acetaldehyde, alkanes, amines, benzenes, pentane, sulfur compounds, and many others (12). After endotoxic administration in the rat, compounds like propanol, butanal, acetophenone, butandiol, hexanone, dimethylether, pentafluoropropionamide, xylene, propanol, toluene, and others have been proposed to serve a multivariate diagnostic analysis of shock versus control animals (13). Alcohols and ketones (e.g., propanol, pentanone, acetone, hexanone) were reported to decrease over a 6 h period in cecal ligation and endotoxin shock but not in hemorrhagic shock (14). In a study with Wiggers hemorrhagic shock 16 different VOCs changed in shock compared with controls, five of which had higher intensities, nine had lower intensities during hypotension and 9 h thereafter, and two increased only during the hypotensive period (15). It is largely uncertain what mechanisms are involved in the formation of VOC and whether their detection may serve as a diagnostic tool to evaluate the effectiveness of therapy. This is the objective of the current report.

Our increasing evidence suggests that pancreatic digestive enzymes in the small intestine and digestive fragments they produce may play a central role in the development of organ failure by escape from the lumen of the intestine after breakdown of the mucosal barrier (16). As digestive enzymes enter into the wall of the intestine, they break down the mucosal lining (17) and start an autodigestion process, which leads to multi-organ failure and death if uninterrupted (18). During this process not only the food content of the intestine, as is the case during normal digestion, but also autologous tissue is degraded to the point that morphological features associated with the intestinal mucosa are destroyed. The process starts at the tip of villi in the small intestine already within minutes of an ischemic insult (19) and can lead to complete destruction of the villi (20–23).

Introduction of ischemia causes formation of VOCs detected by gas chromatography (24). But their source in the tissue remains uncertain. We hypothesize that in experimental peritonitis shock distinct VOCs are generated during proteolytic degradation of the small intestine and are detectable in the breath. Accordingly, we present measurements of VOCs before and after peritonitis shock without and with blockade of the pancreatic proteases in the lumen of the intestine.

## METHODS

### Animals

The animal protocol was reviewed and approved by the UC San Diego Animal Subjects Committee.

Young mature Wistar rats (age 15–20 weeks, 350 g–450 g body weight, Harlan Sprague Dawley) were maintained on a regular diet (Harlan Teklad Rodent diet (W) 8604, 0.29% sodium by weight) without restriction and water *ad libitum*. After general anesthesia (pentobarbital sodium, 50 mg/kg, Abbott Laboratories, Chicago, Ill), the body temperature was maintained at 37°C by keeping the animals on a water-heated support and by the use of a heat blanket. At the end of the 6-h observation period the animals were euthanized (120 mg/kg, Beuthanasia-D Special, Merk Animal Health).

### Peritonitis shock

In this shock model, cecal material (900 mg/kg derived from a mixture of multiple donor rats, diluted by addition of 1 mL of 5% glucose saline solution to 1 g cecal material) was injected into the peritoneal cavity and gently spread by mild skin compression over the abdomen. Control animals without peritonitis were administered the glucose saline solution without cecal material.

### Digestive enzyme blockage

The digestive enzymes were blocked at 60 min after cecal material administration as described (16). Briefly, to block digestive enzymes in the lumen of the intestine, a mid-line incision (2.5 cm) was made to temporarily expose the small intestine. Tranexamic acid (127 mM, Pfizer Inc, New York City, NY) dissolved in GoLYTELY (60 g/L PEG-3350 and electrolytes for oral solution; Braintree Laboratories Inc, Braintree, MS) was injected with a needle (BD Sub-Q 26G 5/8, Becton Dickinson & Co) into the lumen of the intestine from the duodenum to the cecum. Untreated shock controls received GoLYTELY without the protease inhibitor. In a 350 g rat a total fluid volume of 15 mL (at 39°C) was injected into the lumen of the small intestine spaced over eight injections in intervals of about 5 cm and 2 mL was injected into the cecum. The intestine was returned into the peritoneal cavity and the incision closed by sutures.

In addition to the enteral protease inhibition, a mixture was also administered as lavage into the peritoneum composed of tranexamic acid (2 mL; 127 mM dissolved in saline), an antibiotic (ciprofloxacin, 10 mg/mL, Hospira Inc, Lake Forrest, Ill), and albumin (40 mg/mL, Sigma-Aldrich Co, St. Louis, Mo) at 1 h after the placement of cecal material. The mixture serves to inhibit not only digestive enzymes, but also bacteria and lipid cytotoxic mediators (e.g., unbound free fatty acids) that were administered by the cecal material into the peritoneum. The untreated peritonitis shock group received the same volume of saline into the peritoneum and the same volume of GoLYTELY into the lumen of the intestine but without additives. To determine the role of the enteral treatment in VOC generation, we also studied a group of shock rats (n = 3) with peritoneal treatment only (using the same mixture) but without enteral treatment.

### Breath analysis

To collect exhaled air, a tracheotomy was carried out for the placement of a two-way non-rebreathing valve. Exhaled breath samples were collected in Tedlar gas sample bags (1 L, Jensen Inert, Coral Springs, Fla) placed at the end of the tracheostomy tube. A two-way non-rebreathing valve (series 2300 T-shape; Hans Rudolph Inc, Shawnee, Kans) was used for collection of exhaled air into gas sampling bags. Control breath samples were collected before laparotomy to block digestive enzymes. Room air samples were collected as controls for inspired air. Samples were collected in intervals of 30 min over the first hour and then hourly for 6 h.

The gas sample bags were kept at room temperature and analyzed within a period of 2 h after collection. Repeat measurements of the same air sample over a period of 7 days showed that there was no significant loss of VOCs in the bags, for example due to absorption to the Tedlar bags.

The presence of VOCs was detected by use of gas chromatography (Z-Nose Ultra-Fast GC, Model 4200, MicroSense 5.44 GC analysis software, Electronic Sensor Technology, Newbury Park, Calif). The settings on the gas chromatography were standardized (sensor temperature 30°C, column temperature 40°C, valve temperature 140°C, inlet temperature 200°C, trap temperature 250°C, flow 3.0 ccm—helium carrier gas; sample time = 30 s; temperature ramp = 200°C at 5°C/s). Before measurements, the gas chromatography chamber was cleaned by heat (205°C) running helium through the system in a continuous mode until a background was reached with little activity. For the calibration of the Kovach index (KI), an alkane calibration (n-alkane vapors C6-C14; Electronic Sensor Technology) solution was tested five consecutive times. The chromatography profile was digitally scanned and peak amounts were determined with peak-detection software provided with the Z-nose. The values are expressed in the form of “amount” (i.e., digital detector GC output).

A list of 25 VOCs was tested with the same GC settings to determine their associated KI values (acetone; amine; n-butyl acetate; 2-butanone; t-butanol; butyraldehyde; benzaldehyde; cadaverine; carbon disulfide; chlorobenzene; dimethyl sulfide; 1,4-diaminobutane; 1,5-diaminopentane; 1,2-dichloroethane; ethanol; indole; limonene; 6-methyl-5-hepten-2-one; 4-methylphenol; pentanoic acid; 1-propanol; putrescine; methylamine N-oxide, TMAO; o-xylene). The list was derived from literature values for gastrointestinal, respiratory, and cancer patients and associated in part with tissue decay (6, 11, 25–32). Each compound was dissolved in distilled water and the supernatant tested in the GC. The KI values for these compounds were compared with the KI values determined in the breath samples of the rats.

### Statistics

All measurements are summarized as mean ± standard deviation. Repeated measures ANOVA followed by Bonferroni *post-hoc* test was used to compare sham controls with untreated and treated shock groups. Comparison between treated and untreated groups was carried out by Student *t* test. *P* < 0.05 was considered significant.

## RESULTS

**Fig. 1:**
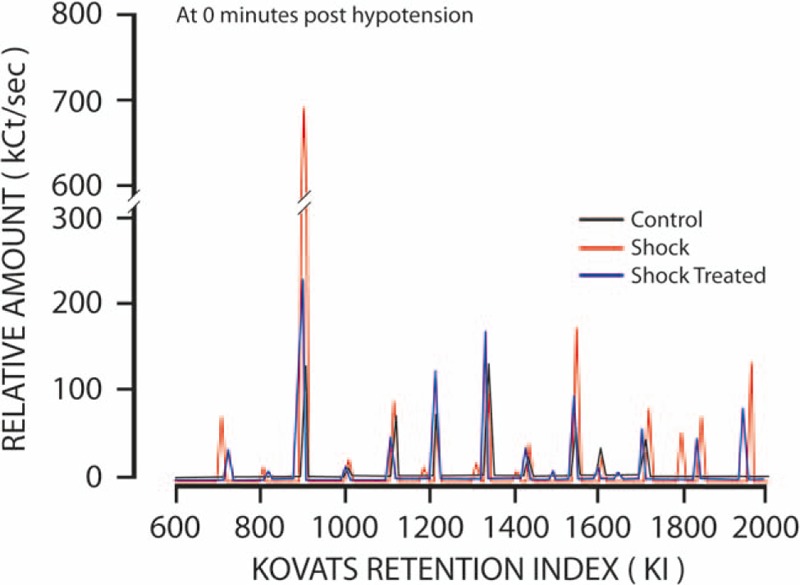
An example of GC profile for control without peritonitis (Control group), untreated peritonitis (Shock group), and peritonitis with treatment by inhibition of the digestive proteases with TXA (Shock *Treated*, see the Methods section).

The control breath samples had a gas chromatography profile that was similar among the control animals with relatively small variation among rats (typical variance about 10%). During peritonitis shock breath samples exhibited shifting peak amounts at distinct KI values, some of the peak amounts increased and some also decreased (Fig. 1).

Note elevation of peak amounts, e.g., at KI 720, 900, and 1,550.

**Fig. 2:**
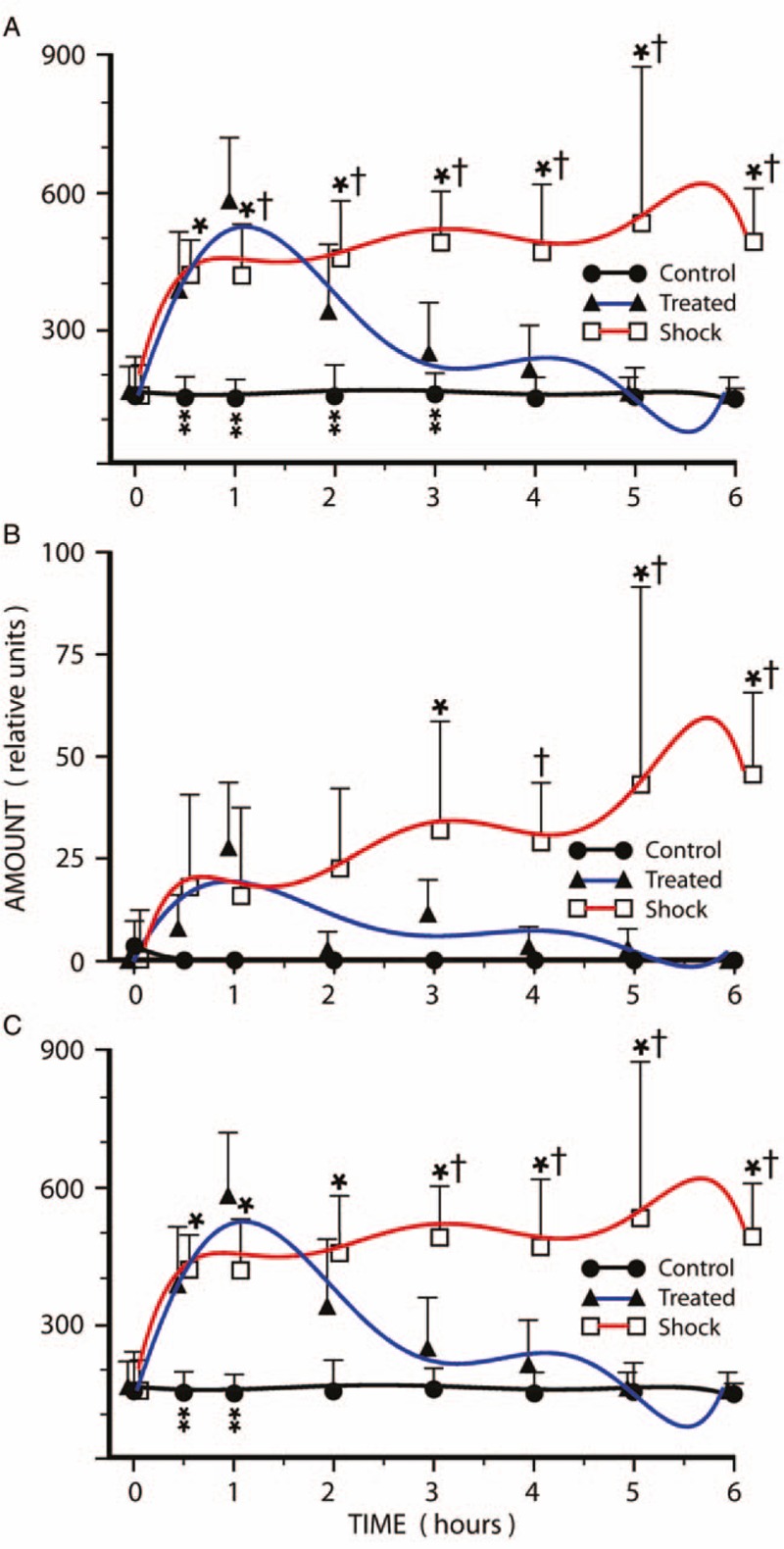
The amounts of VOCs at KI (A) 720, (B) 900, and (C) 1,550 measured over a period 6 h from rat breath samples after exposure to peritonitis shock without (Shock group) and with enteral protease inhibition with TXA (Treated group) at 1 h after initiation of the peritonitis.

Analysis of changes in peak amounts over a range of KI values between about 600 and 2,000 showed that the peak amounts at KI 720, 900, and 1,550 in the breath (Fig. 2) exhibited significant differences between sham control and shock. While in sham controls the peak amounts did not rise significantly over the 6-h period of the current experiments, in peritonitis shock the peak amounts at KI 720, KI 900, and KI 1550 monotonically and significantly rose. The rise occurred already within the first hour of the peritonitis and there was no trend for a reduction of peak amounts over the course of the experiment.

Control represents a sham group without peritonitis. n = 6 to 8 rats in each group. Amounts of VOCs in a separate group (n = 3 rats) with peritonitis shock but with treatment in the peritoneum only (with same mixture, see the Methods section) and without enteral treatment gave values that were not significantly different compared with the untreated Shock group. At 6 h the amounts of VOCs in this group with peritoneal treatment only at KI 720, 900, and 1,550 were 315 ± 43, 117 ± 34, 483 ± 61, respectively. ^∗^*P* < 0.05 Control versus Shock group; ^†^*P* < 0.05 Shock versus Treated group; ^∗∗^*P* < 0.05 Control versus Treated group. KI indicates Kovach index; VOC, volatile organic compounds.

In contrast, after the combined enteral/peritoneal treatment and blockade of the digestive enzymes with tranexamic acid at 1 h, the peak amounts started to decrease and after 6 h fell to values that were not different compared with non-shock controls (Fig. 2). The decreases in peak amounts were only observed after treatment. The *enteral* treatment is essential for the reduction of the VOC amounts. Without enteral administration and only peritoneal treatment in shock animals, there was no reduction of the peak amounts and their values were significant difference compared with the untreated peritonitis group (Fig. 2, legend).

Peak amounts at KI values, other than at 720, 900, and 1,550, exhibited no significant changes in the current exhaled gas analysis, although several peak amounts reached close to significant values.

Among the set of VOCs we tested (see the Methods section), 1,4-diaminobutane (Lot# STBC1987 V, SIGMA-Aldrich Co) had a KI at 720 ± 5 and trimethylamine N-oxide (TMAO, 317594, SIGMA-Aldrich Co) a KI of 900 ± 5. We could not find in this list a compound that had a peak amount at KI 1550. All other VOC peak amounts fell at least by a value of ± 50 outside the KI values observed in the breath samples. Both 1,4-diaminobutane and TMAO have a strong foul odor.

## DISCUSSION

The current results indicate that in experimental peritonitis the breath contains within less than an hour elevated levels of VOCs over several hours that can be inhibited by enteral protease inhibition in the small intestine, but not without it. These measurements are in line with qualitative observations during a previous study with an identical peritonitis shock model (16) as well as other forms of shock (including hemorrhagic and endotoxic shock) in which we noticed foul volatile odor originating from the small intestine. Without exception (18 out of 18 rats examined) the odor was noticed in those animals that succumb to the insult, but less so in surviving animals. In addition to mortality the presence of the odor was accompanied by escape of digestive proteases into the wall of the intestine, lesion formations in the small intestine and the lung. All animals in these studies were maintained with food and water *at libitum* at the time before shock. Therefore, the small intestines were in part filled with food items so that the pancreatic digestive enzymes in the partially digested food and/or the wall of the intestine could serve as a source of the VOCs (16).

A combination of enteral and peritoneal placement of tranexamic acid, a serine protease inhibitor, e.g., for pancreatic trypsin (17), served to reduce the VOC amounts. The protection provided by inhibition of the digestive proteases in the intestine and peritoneum includes beside a reduction of the mortality also a significant reduction of the lesions in the small intestine, heart, and lung, including a reduction of the protease activity in the wall of the small intestine and in plasma. Inhibition of the pancreatic digestive enzymes in hemorrhagic shock also reduces the development of insulin resistance by proteolytic cleavage of the ectodomain of the insulin receptor (33). This evidence suggests that the powerful digestive enzymes in the small intestine may be a major producer for the VOCs that eventually appears in expired air. The VOCs appear to be carried in the plasma and diffuse into the airways.

The VOCs found in this study could be detected without the use of a chemical concentrator during sample collection. They are in sufficient concentrations to be detectable by unaided human smell sensor. Among specific VOCs with equal KI values as seen in the breath samples were 1,4-diaminobutane, TMAO, and a volatile compound whose identity remains to be identified. TMAO is a foul-smelling compound with below body temperature melting points. It is a metabolite from dietary phosphatidylcholine (e.g., lecithin) and can be generated by certain intestinal bacteria (e.g., by generation of trimethylamine that can be transformed into TMAO) (34). Elevated levels of TMAO have been associated with cardiovascular risk (25, 35).

The mechanism by which the TXA protease inhibition blocks the formation of TMAO, as seen in the current experiments, remains to be identified. TXA was administered enterally and by lavage into the peritoneal cavity around the intestine. The fact that it serves to acutely reverse VOCs formation indicates that their formation is ongoing and protease dependent. The fact that the peritoneal without enteral blockade is inadequate to block the formation of the VOCs (Fig. 2, legend) suggests that the digestive enzymes in the lumen of the small intestine are centrally involved. A source for the odor may be food material in the lumen of the intestine, as seen in fecal material (12), but could also include intestine itself, since digestive enzymes enter the wall of the intestine in shock. TXA serves to preserve the mucosal barrier. If a source of the foul-smelling VOCs is in the lumen of the intestine, then the preservation of the barrier my reduce entry of the VOC into the circulation and into the breath. If the source of the VOCs is in the wall of the intestine, the mode of action by TXA is to prevent proteolytic degradation of intestine and also other tissues into which digestive protease leak during peritonitis shock.

TXA is recognized and marketed as an antifibrinolytic agent, able to block formation of plasmin when administered i.v. But its actions as protease inhibitor appear to be broader. In the current application it was used to block serine proteases (e.g., trypsin) in the lumen of the small intestine without i.v. infusion. Enteral blockade of digestive enzymes serves to attenuate a variety of tissue injuries, organ dysfunctions, and failures in multiple models of shock (16, 33), and thus the current observation for attenuation of VOCs is in line with the evidence for other forms of protection from digestive enzymes.

We consistently smelled VOCs from the intestine in non-surviving animals that were subject to hemorrhagic shock, a model without the use of cecal material with bacteria or an antibiotic agent in the peritoneum. While measurements of VOCs with and without antibiotic treatment (as compared with enteral protease inhibition) in shock remain to be carried out, this evidence suggests that VOCs can be formed without a major bacterial load.

Key questions to be investigated are whether VOCs in shock patients detected by an electronic breath sensor are similar to those seen in experimental models and if present whether they serve to predict impending organ failure before it reaches an irreversible stage associated with actual organ failure. The current evidence indicates that VOC generation is an early event in a peritonitis induced shock model but the amounts detected in the breath may depend on the extent of intestinal damage. In the current model the major length of the small intestine wall tissue is subject to infiltration by digestive enzymes (16) and a potential source for VOCs. If in other situations shorter segments of the small intestine become ischemic and are infiltrated by digestive enzymes, the magnitude of the VOCs detected in the breath may be lower. This situation in ICU patients is largely unknown and will require clinical measurements from the time of earliest access to breath samples.
